# In vitro modulation of Schwann cell behavior by VEGF and PDGF in an inflammatory environment

**DOI:** 10.1038/s41598-021-04222-7

**Published:** 2022-01-13

**Authors:** Souptik Basu, Indra N. Choudhury, Lynn Nazareth, Anu Chacko, Todd Shelper, Marie-Laure Vial, Jenny A. K. Ekberg, James A. St John

**Affiliations:** 1 Clem Jones Centre for Neurobiology and Stem Cell Research, Griffith University, Nathan, QLD Australia; 2 Menzies Health Institute Queensland, Griffith University, Southport, QLD Australia; 3 Griffith Institute for Drug Discovery, Griffith University, Nathan, QLD Australia

**Keywords:** Spinal cord injury, Schwann cell

## Abstract

Peripheral glial cell transplantation with Schwann cells (SCs) is a promising approach for treating spinal cord injury (SCI). However, improvements are needed and one avenue to enhance regenerative functional outcomes is to combine growth factors with cell transplantation. Vascular endothelial growth factor (VEGF) and platelet-derived growth factor (PDGF) are neuroprotective, and a combination of these factors has improved outcomes in rat SCI models. Thus, transplantation of SCs combined with VEGF and PDGF may further improve regenerative outcomes. First, however, we must understand how the two factors modulate SCs. In this in vitro study, we show that an inflammatory environment decreased the rate of SC-mediated phagocytosis of myelin debris but the addition of VEGF and PDGF (alone and combined) improved phagocytosis. Cytokine expression by SCs in the inflammatory environment revealed that addition of PDGF led to significantly lower level of pro-inflammatory cytokine, TNF-α, but IL-6 and anti-inflammatory cytokines (TGF-β and IL-10), remained unaltered. Further, PDGF was able to decrease the expression of myelination associated gene Oct6 in the presence of inflammatory environment. Overall, these results suggest that the use of VEGF and/or PDGF combined with SC transplantation may be beneficial in SCI therapy.

## Introduction

Spinal cord injury (SCI) has a consequential impact on individuals, families and societies^1^. The worldwide incidence is estimated to be 13.1–220.0 cases per million people, combining developed and developing nations^2^. After the initial injury, the SCI progresses through acute, secondary and chronic stages, typically initially worsening and then stabilizing^3^. A multitude of strategies have been implemented for therapeutic interventions in SCI^4–6^ with most targeting secondary injury stage to limit fibrotic scar formation^7^. One such promising approach involves the transplantation of Schwann cells (SCs) which are peripheral glial cells^8–10^. SCs have several favourable properties that make them potentially suitable for neural repair therapies, including the ability to phagocytose myelin and cell debris, to recruit macrophages and to aid axon regeneration via physical and neurotrophic support. Although SC transplantation has been used to improve motor recovery in adult rat SCI models^11,12^, the limited efficacy of this approach as well as the complex immunological and cellular profile of SCI suggest that improvements could be achieved using combinatorial approaches^13^.

Vascular endothelial growth factor (VEGF, sometimes termed VEGF-A) is a well-known dimeric angiogenic factor responsible for promoting blood vessel growth, increasing vascular permeability and for promoting cell survival^14^. Over the last decade, VEGF has been reported to also be neuroprotective, and reduced levels of VEGF is linked with neurodegenerative disorders^15,16^. As a peripheral nerve injury therapy, application of VEGF has been shown to be beneficial for rat sciatic nerve regeneration, promoting both invasion of SCs into the injury site and neovascularisation^17^. VEGF has also been shown to modulate central nervous system (CNS) glia (astrocytes and microglia). VEGF promotes proliferation and motility of both glial types, in addition to improving gap junction intercellular communication in astrocytes^18,19^. When exogenously administered, VEGF has been shown to protect recovering neurons in ischaemic injury^20^ by regulating outward delayed rectifier potassium channels^21^ and reducing caspase 3 activities associated with cell death pathways^22^. In addition to its neuroprotective role, VEGF is also reported to possess immunomodulatory functions where it has been shown to help in monocyte recruitment and lead to arteriosclerotic formation in animal models^23^.

Another growth factor related to VEGF, platelet-derived growth factor (PDGF), plays an important role in blood vessel maturation by recruiting and promoting differentiation of pericytes^24^. PDGF is dimeric and can consist of any combination of A and B subunits (AA, BB, AB). The neuroprotective role of PDGF has been linked with suppression of hydrogen peroxide-induced caspase 3 activation^25^. In brain ischaemic stroke rat models, PDGF has been shown to increase expression of various neurotrophins, such as nerve growth factor (NGF) and neurotrophin-3 (NT-3), in pericytes^26^. Similar to VEGF, PDGF has also been reported to have immunomodulatory function, by increasing astrocyte-mediated secretion of monocyte chemoattractant protein-1 (MCP-1) in an in vitro human model of HIV-associated neurocognitive disorder^27^. Thus, the role of both VEGF and PDGF in neuroprotection as well as in immunomodulation suggest that they may be effective when used in a combinatorial treatment regimen for nervous system injuries.

In this context, when a combination of VEGF and PDGF was administered in a rat cortical injury model^28^, it resulted in delayed astrocyte activation around the lesion (thus, delayed formation of the glial scar), as well as delayed infiltration by ramified microglia, accompanied by an increase in phagocytic microglia (i.e. modulation of the immune response). These changes occurred only in the short-term (2–5 days post simultaneous injury and VEGF/PDGF administration), but encouragingly were accompanied by enhanced axonal sprouting^28^. Combined administration of VEGF and PDGF has also been shown to promote regeneration of the olfactory nervous system. After unilateral bulbectomy, a common olfactory nervous system injury model involving removal of one olfactory bulb, which is the target region in the forebrain for axons of sensory olfactory neurons^29^, combined VEGF and PDGF treatment was shown to stimulate regeneration of sensory olfactory neurons in mice^30^.

Studies have also investigated the potential for using combined VEGF and PDGF treatment in SCI models. In rats with SCI, combined treatment with VEGF and PDGF has been shown to reduce secondary degeneration^31^ and improve locomotor functions^32^.

Two key impeding factors that obstruct regeneration after SCI are (1) accumulation of myelin-derived debris and (2) the hostile, pro-inflammatory milieu at the injury site^33^. An effective therapeutic strategy will need to target these various barriers. It is possible that the transplantation of SCs can aid the phagocytic clearance of debris^34,35^ and the combined application of VEGF and PDGF can modulate the inflammatory environment. However, before an in vivo study can be undertaken, we need to determine whether VEGF/PDGF in vitro can (1) affect SC phagocytosis of myelin debris in a pro-inflammatory environment, (2) promote a congenial neuroprotective environment by reduction of pro-inflammatory cytokines and increase of anti-inflammatory cytokines, and (3) promote appropriate expression of nerve repairing and myelin expressing genes. Our study showed that both VEGF and PDGF can combine to potentiate SC responses and has the potential to be enhance outcomes for SCI repair.

## Results

### Effects of VEGF and PDGF on SC proliferation

**Figure 1:**
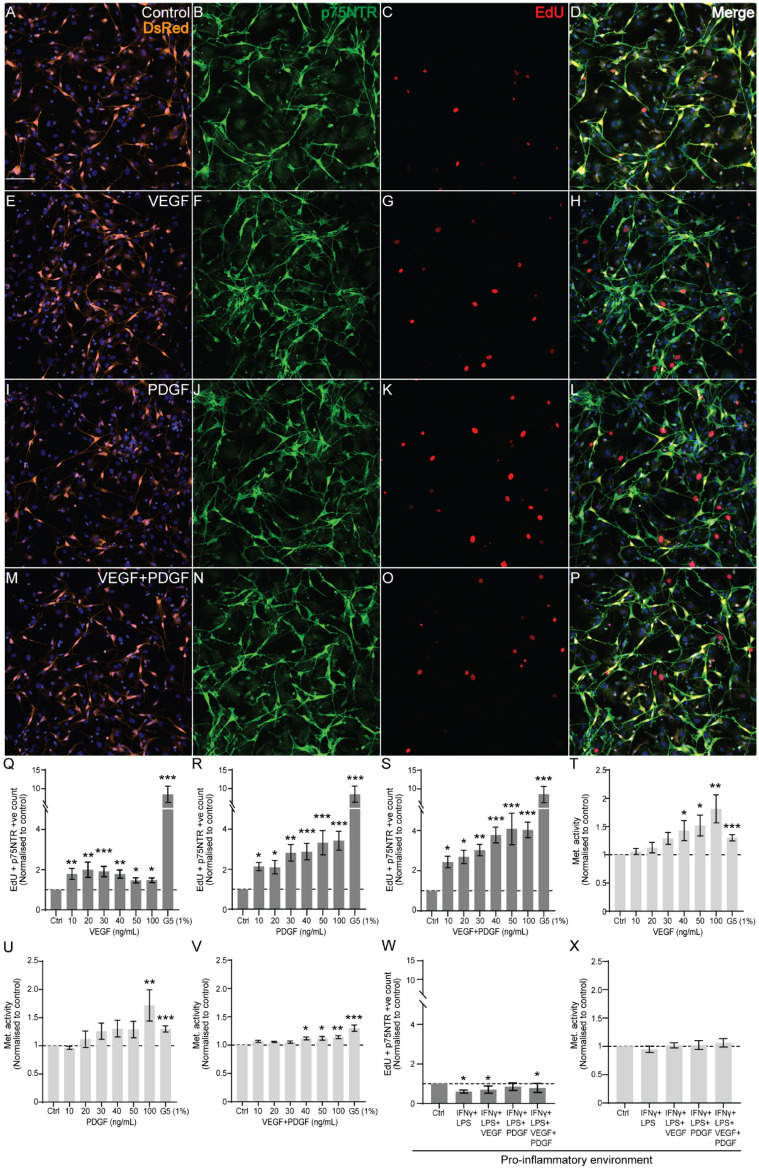
Effect of VEGF, PDGF on cell counts and viability of SCs. (**A–P**) Confocal images show examples of DsRed-expressing (orange), p75NTR (green) and EdU positive (red) SCs treated for 24 h with (**A–D**) medium alone (control), (**E–H**) VEGF, (**I–L**) PDGF and (**M–P**) Combined VEGF and PDGF. Blue: nuclei (Hoechst stain). Scale bar: 100 µm. (**Q–S**) EdU and p75NTR positive cell counts per fields of view (FOV) in relation to control (medium alone, set to 1) for cells treated with different concentrations of (**Q**) VEGF, (**R**) PDGF and (**S**) VEGF + PDGF. (**T–V**) Viability (assessed by resazurin assay) of SCs treated with (**T**) VEGF, (**U**) PDGF and (**V**) VEGF + PDGF. (**W, X**) Effects of VEGF and PDGF on SC proliferation in a pro-inflammatory environment (1 ng/mL IFN-γ + 100 ng/mL LPS); (**W**) EdU and p75NTR positive cell counts; (**X**) Metabolic activity (resazurin assay). Dashed lines represent normalisation of EdU count to control (set at 1). * p ≤ 0.05, ** p ≤ 0.01, *** p ≤ 0.001 measured using one way ANOVA with post hoc Dunn’s test. Error bar represents mean ± SEM for three biological replicates.

Our first aim was to determine how VEGF and PDGF affected proliferation and metabolic activities of SCs, both individually and in combination. Previously, both VEGF^36^ and PDGF^37^ have been shown to promote proliferation of rodent SCs, however, the effects of the two factors in combination on SC proliferation is not known. Primary mouse SCs were obtained from S100β-DsRed transgenic mice (SCs express the marker DsRed protein). In these cultures, > 80% of cells were DsRed positive and 61.2% were immunostained with anti-p75NTR antibodies (Fig. 1). The cells were cultured in the absence (control) (Fig. 1A-D) and presence of VEGF (Fig. 1E-H), PDGF (Fig. 1I-L) and combined VEGF and PDGF (Fig. 1M-P). We also included a positive control, glial supplement G5, which contains a mixture of numerous factors and is well documented to stimulate glial proliferation^35,38^.

To assess proliferation, we added EdU to the cells which gets incorporated into newly synthesized DNA. Further, co-staining with antibodies against p75NTR identified SCs (although not all SCs express p75NTR as they may be in different stages of differentiation or maturity). Our observations showed that addition of VEGF and PDGF led to significant proliferation of SCs. While VEGF showed better outcome at an earlier dose (30 ng/mL) similar to G5 (Fig. 1Q), PDGF demonstrated a similar pattern at higher doses (40–100 ng/mL) (Fig. 1R). Surprisingly, combined VEGF and PDGF had a further increased effect throughout all the dose combinations (Fig. 1S). Since a considerable amount of the cell population also contained DsRed -ve with p75NTR -ve cells, we also assessed the proliferation of these cells when growth factors were administered (Supplementary Figure S1). We found that the growth factors (single or combined) did not significantly increase the population of these cells, except for combined VEGF + PDGF at 100 ng/mL. Interestingly, VEGF at doses 30–50 ng/mL significantly decreased the population of the cells.

We also assessed viability using the resazurin assay which measures the metabolic activity of cells—as only viable cells are metabolically active; the assay is commonly used for assessing cell viability. In accordance with the results of the cell count assay, VEGF at 50 and 100 ng/mL (Fig. 1T) and PDGF at 100 ng/mL (Fig. 1U) increased viability of the heterogenous population to a level similar to that in the presence of the G5 supplement. Similarly, a combination of VEGF and PDGF at a range of concentrations (40–100 ng/mL) significantly stimulated viability as assessed by the resazurin assay (Fig. 1V). To maintain a controlled SC growth environment and based on previously established studies regarding the effects of VEGF^36^ and PDGF^39^ on neural cells, we used a concentration of 50 ng/mL of VEGF and PDGF (alone and in combination) for further assays.

A nerve injury site, in the CNS (such as a SCI site), is initially a pro-inflammatory hostile environment. We therefore also assessed the effects of the two factors in an in vitro model of inflammation (IFN-γ + LPS) which has been used previously^40^. Although, the pro-inflammatory environment itself reduced the growth of SCs (Fig. 1W), it did not reduce its viability (Fig. 1X). Additionally, in this environment, both VEGF and combined VEGF and PDGF resulted in a significantly lower proliferation of SCs compared to control (Fig. 1W). Surprisingly, PDGF provided protection to SCs by maintaining cell count and viability. Thus, VEGF and PDGF (single and combination) can stimulate SC proliferation, but in a pro-inflammatory environment, PDGF is beneficial to the SCs.

### Inflammation impairs SC-mediated clearance of myelin debris but PDGF stimulates clearance

The presence of myelin debris after SCI can exacerbate inflammation and impair regeneration^41^. Therefore, promoting myelin clearance from the site would aid regeneration. As stimulation of glial-mediated phagocytosis has been suggested to improve transplantation outcomes^42,43^, we therefore next investigated whether VEGF and/or PDGF could modulate the phagocytic activity of SCs. Previous in vivo studies have shown that SCs contribute to myelin debris clearance both by autophagy and phagocytosis^34^ and can phagocytose both myelin and necrotic bodies in vitro^35^. Clearance of myelin by SCs in an inflammatory in vitro environment has, however, not previously been reported. Before assessing the effects of the growth factors, we therefore investigated how an inflammatory environment affected SC-mediated phagocytosis of myelin. We exposed mouse primary SCs to pro-inflammatory factors (IFN-γ and LPS as described in the previous section) and determined the effects of the pro-inflammatory environment on internalization of myelin debris into acidic organelles.

**Figure 2:**
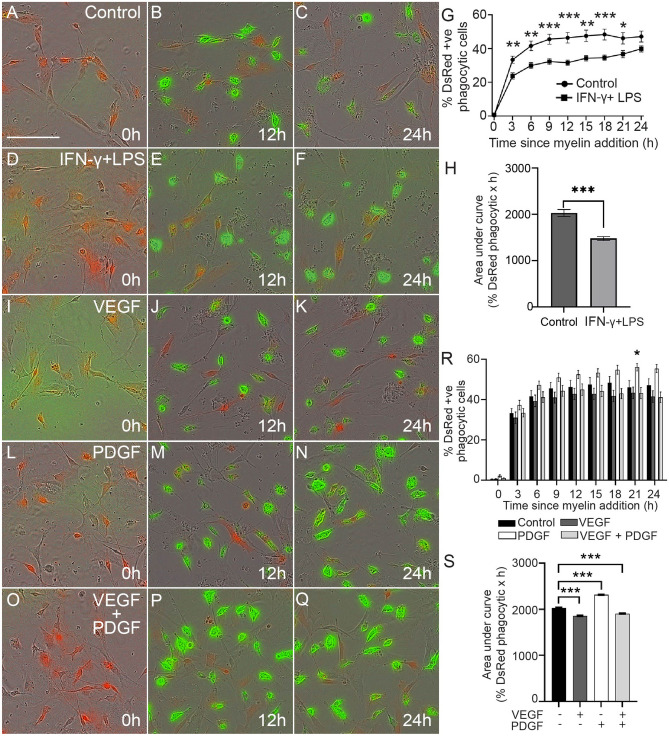
Effects of inflammatory mediators and growth factors on myelin phagocytosis by SCs. (**A–F**) Representative time-lapse images at 0, 12 and 24 h post myelin exposure; images show DsRed-expressing SCs (red) with internalized myelin debris (green). (**A–C**) control, (**D–F**) inflammatory environment (IFN-γ** + **LPS). (**G**) Graph showing the percentage of DsRed SCs containing myelin debris at different times (3 h intervals) over 24 h, for the control condition and the inflammatory condition (IFN-γ** + **LPS). (**H**) Graph showing total phagocytosis efficacy (AUC for percentage of DsRed SCs containing myelin debris across 24 h) for control cells and cells in the inflammatory condition (IFN-γ** + **LPS). (**I–Q**) Time-lapse images of cells in control medium (no inflammatory mediators) with (**I–K**) VEGF (50 ng/mL), (**L–N**) PDGF (50 ng/mL) or (**O–Q**) VEGF + PDGF (both 50 ng/mL). (**R**) Graph shows percentages of DsRed SCs containing internalized myelin debris over 24 h for the control, VEGF, PDGF and VEGF + PDGF conditions. (**S**) Graph shows total phagocytosis efficacy (AUC over 24 h) for the control, VEGF, PDGF and VEGF + PDGF conditions. * p ≤ 0.05, ** p ≤ 0.01, *** p ≤ 0.001 measured using two-way ANOVA with post hoc Fisher’s LSD test for (**G**) and (**R**); unpaired two-tailed t-test for (**H**) and one-way ANOVA followed by Dunnett’s multiple comparisons test for (**S**). Error bar represents mean ± SEM for three technical replicates of three biological replicates. Scale bar in time-lapse images (shown in **A**): 100 μm.

We first labelled extracted myelin with pHrodo STP dye as previously described^35^ and then challenged the SCs with myelin debris either in the absence (control environment) or in the presence of IFN-γ and LPS (pro-inflammatory environment). The pHrodo STP dye is pH-sensitive and has an increase in fluorescent emission below pH 6.0, and hence after the myelin debris is internalized into acidic organelles (lysosomes/phagolysosomes) it exhibits fluorescence (green). For the following experiments, we set a low threshold cut-off level to enable detection when the pHrodo was just starting to fluorescence and thus the quantification reflects mainly the processing of myelin and myelin digestion, but also some level of phagocytosis. The cells were continuously imaged over 24 h, during which the appearance of fluorescent debris in acidic organelles was tracked. We observed that SCs internalized and processed the myelin as early as 30 min post-administration of myelin, with internalized myelin being detected in cells for up to 24 h in both the control (Fig. 2A-C) and pro-inflammatory environment conditions (Fig. 2D-F). To confirm that the myelin debris was engulfed and present inside cells, we used confocal microscopy and 3D rendering of z-stacks (Supplementary Fig. S2). We analyzed the number of DsRed-expressing SCs which exhibited pHrodo-labelled myelin (green) and determined the percentage of SCs that had internalized myelin debris into lysosomes/phagolysosomes. In these cultures, SCs expressed DsRed protein and p75ntr (Fig. 1), however there cells (15–20%) that did not express DsRed or p75ntr and were likely to be mainly fibroblasts, as shown previously^35^, therefore, we only analyzed cells that expressed DsRed.

SCs in the inflammatory environment phagocytosed significantly less myelin debris than SCs in control conditions over 3–21 h (Fig. 2G). However, the percentage of cells containing myelin in the control condition plateaued from 9 h onwards, while the cells in the inflammatory condition continued to increase myelin internalization into acidic organelles after this time. By 24 h, there was no significant difference between the two groups. Thus, the pro-inflammatory mediators decreased the rate by which SCs internalized the myelin into acidic organelles.

To determine the efficacy of internalization of myelin over the entire 24 h assay by SCs, we also determined the area under curve (AUC) according to our previously published protocol^35^. We found that the SCs in the inflammatory condition were significantly less efficient in internalizing myelin debris into lysosomes/ phagolysosomes than cells in the control condition (Fig. 2H).

We next tested whether VEGF (Fig. 2I-K) and PDGF (Fig. 2L-N), alone or in combination (Fig. 2O-Q) could modulate the phagocytic activity of SCs in a control non-inflammatory environment. At the individual time-points, VEGF and/or PDGF did not affect SC-mediated myelin phagocytosis, except for PDGF at 21 h which exhibited a higher percentage of cells with internalized myelin than the control (Fig. 2R). However, there appeared to be a trend toward higher phagocytosis by cells treated with PDGF at each time, and lower phagocytosis for the other treatments. To assess this, we looked at the overall efficacy (AUC over 24 h) of phagocytosis of myelin debris, PDGF provided significant internalization while VEGF and combined VEGF + PDGF suppressed the activity in comparison to control (Fig. 2S). Thus, our observation led us to the conclusion that PDGF could enhance overall clearance of myelin debris by SCs.

**Figure 3:**
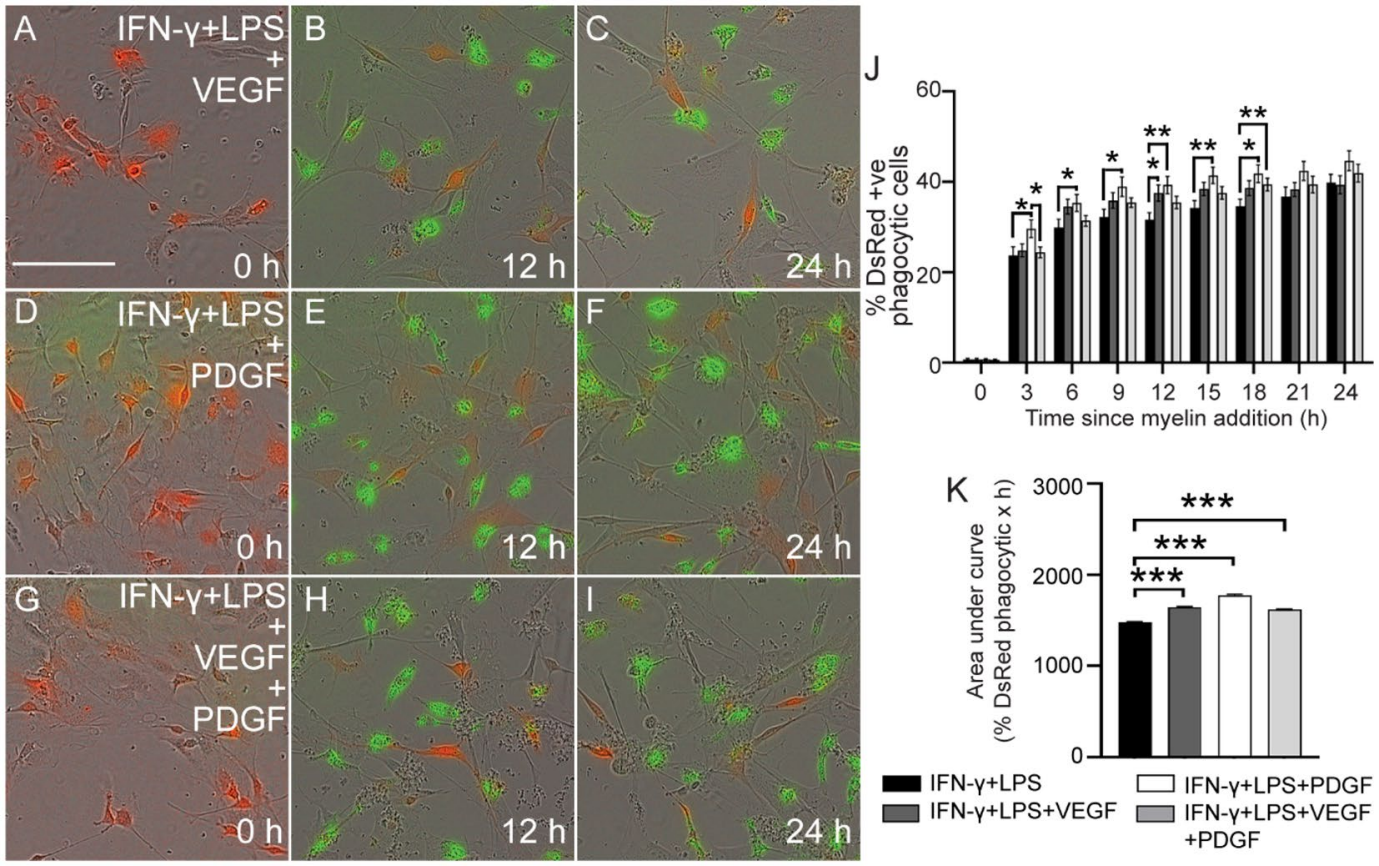
Phagocytosis of myelin debris by SCs in inflammatory conditions co-stimulated with growth factors. (**A–I**) Time lapse images of DsRed SCs (red) phagocytosing myelin debris (green) at 0, 12 and 24 h. Shown are representative time-lapse images for SCs in the pro-inflammatory condition with (**A–C**) VEGF, (**D–F**) PDGF and (**G–I)** VEGF + PDGF. Scale bar: 100 μm. (**J**) Graph shows the percentages of SCs containing internalized myelin debris over 24 h in the presence of inflammatory mediators only (IFN-γ** + **LPS), or inflammatory mediators combined with VEGF, PDGF or VEGF + PDGF. * p ≤ 0.05, ** p ≤ 0.01; two-way ANOVA with Fisher’s LSD post hoc test. (**K**) Total phagocytic efficacy (AUC for 24 h) for the same groups as described in (**J**). Colour of the bars represent the variable conditions in both (**K**) and (**J**). *** p ≤ 0.001; one-way ANOVA with Dunnett’s multiple comparisons test. Error bar represents mean ± SEM for three technical replicates of three biological replicates.

We next assessed whether the two growth factors could stimulate SC-mediated phagocytosis of myelin in a pro-inflammatory environment (mimicking an injury site). Thus, VEGF, PDGF and VEGF + PDGF were administered to cells together with the pro-inflammatory mediators (IFN-γ + LPS) (Fig. 3A-I). At individual time-points, PDGF significantly stimulated phagocytosis over a long time-course (3–18 h) compared to phagocytosis in the presence of inflammatory mediators alone (IFN-γ + LPS only) (Fig. 3J). VEGF alone or in combination with PDGF did not significantly alter phagocytosis except at certain time-points such as 12 h for VEGF and 18 h for combined VEGF + PDGF (Fig. 3J). However, again, there appeared to be a trend toward higher phagocytosis at each time point so we analysed the overall phagocytic efficacy (AUC during 24 h). This revealed that all three growth factor conditions significantly stimulated phagocytic efficacy, with PDGF eliciting the strongest effect (Fig. 3K). Thus, these growth factors, particularly PDGF, can stimulate SC-mediated myelin phagocytosis in a pro-inflammatory environment.

### PDGF reduces secretion of pro-inflammatory cytokines by SCs during inflammation.

As we suggest that a combination of transplanted SCs and growth factors such as VEGF and PDGF may be a potential treatment option for SCI, we need to first understand how an inflammatory environment affects the cytokine profile of SCs. We also need to determine potential effects of VEGF and PDGF on SC-mediated cytokine secretion and finally the effect of these growth factors in an inflammatory environment. We, therefore, next analyzed the cytokine production (at transcriptional and translational levels) by SCs incubated in control medium and in an inflammatory environment (IFN-γ + LPS). We assessed two pro-inflammatory cytokines, TNF-α^44^ and IL-6^35^, as well as two anti-inflammatory cytokines: IL-10^44^, TGF-β^45^.

**Figure 4:**
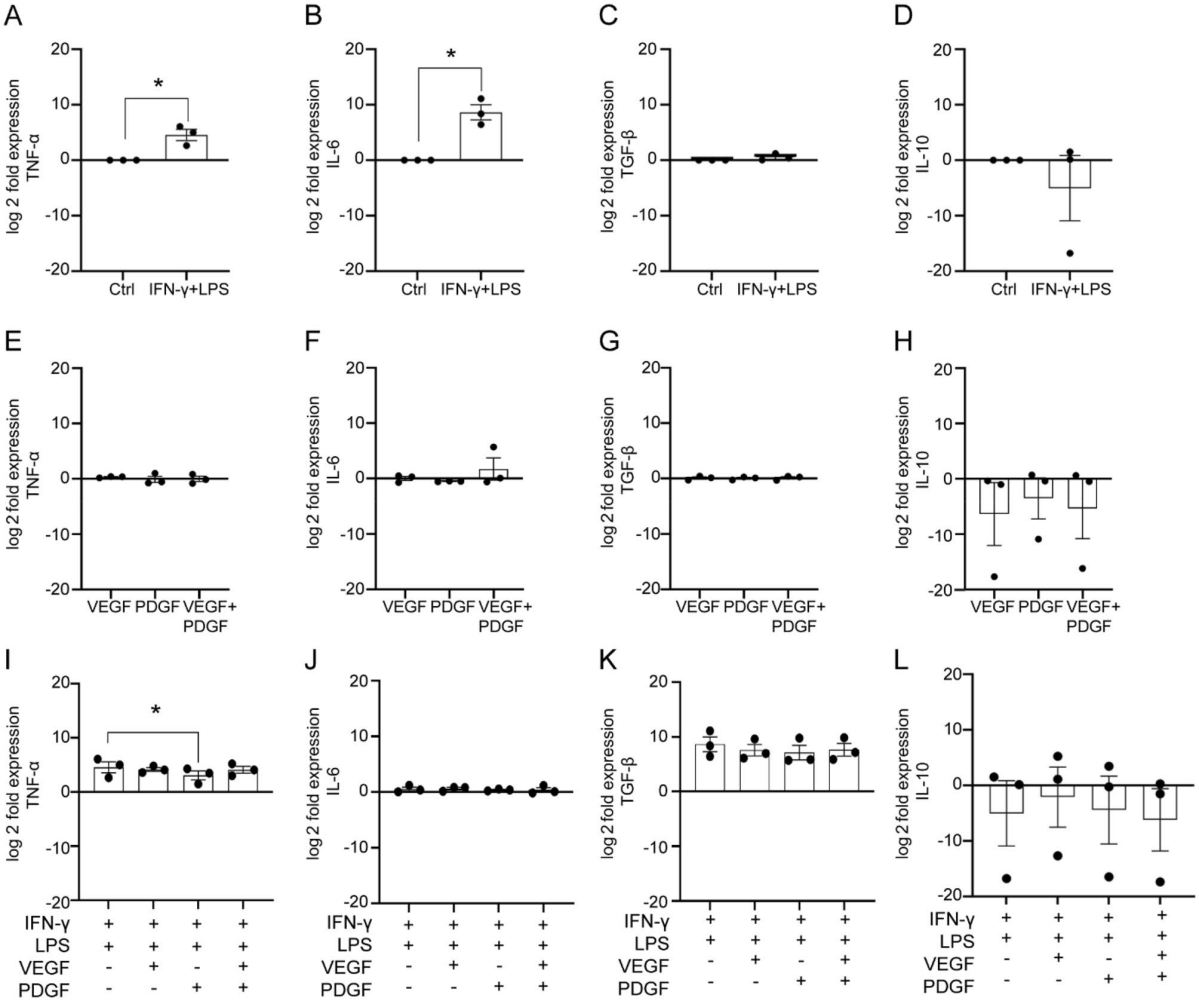
qPCR analysis of effects of VEGF and PDGF on cytokine gene expression in SCs in inflammatory and non-inflammatory conditions. (**A–D**) Effect on cytokine gene expression in control and inflammatory condition (IFN-γ** + **LPS). Bar graphs representing log twofold expression comparison of cytokine genes: TNF-α (**A**), IL-6 (**B**), TGF-β (**C**) and IL-10 (**D**). (**E–H**) Effect of VEGF and/or PDGF on cytokine gene expression (without inflammatory condition). Bar graphs representing log twofold expression comparison of cytokine genes: TNF-α (**E**), IL-6 (**F**), TGF-β (**G**) and IL-10 (**H**). (**I–L**) Effect of VEGF and PDGF on cytokine gene expression in the presence of inflammatory condition. Bar graphs representing log twofold expression comparison of cytokine genes: TNF-α (**I**), IL-6 (**J**), TGF-β (**K**) and IL-10 (**L**). * p ≤ 0.05, measured using unpaired t-test with Welche’s correction for (**A**–**D**) and one way ANOVA followed by post hoc Fisher’s LSD test for (**E**–**L**). Error bar represents mean ± SEM for three biological replicates.

We used real time quantitative PCR (qPCR) to assess how the growth factors affected the cytokine profile of SCs at the transcriptional level, in the absence or presence of an inflammatory environment. First, we analysed the effects on the inflammatory mediators (IFN-γ + LPS) alone on the expression of a range of cytokine genes using qPCR. We found that inflammatory mediators significantly up-regulated the pro-inflammatory genes TNF-α (Fig. 4A) and IL-6 (Fig. 4B) compared to control condition while TGF-β and Il-10 were unaltered (Fig. 4C-D).

Next, we assessed how VEGF and PDGF (alone and in combination) influenced the expression of these genes, in the absence and presence of the added inflammatory mediators. We observed that expression of all the genes remained unaltered (Fig. 4E-H) in the absence of inflammatory mediators. When VEGF and PDGF were added into the inflammatory environment, PDGF significantly lowered expression of TNF-α (Fig. 4I) compared to inflammatory mediators alone. However, the expression of IL-6, TGF-β and IL-10 (Fig. 4J-L) genes were not significantly different.

To complement the gene expressions, cytokine levels were determined using ELISA.

**Figure 5:**
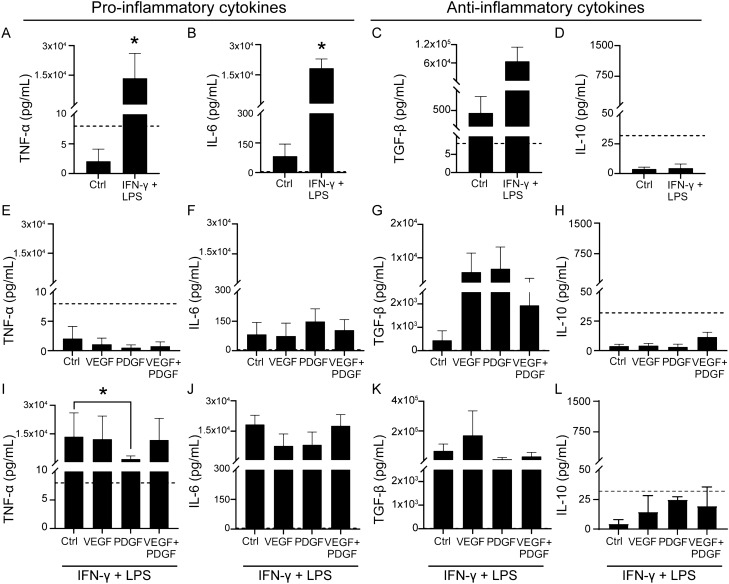
Effects of PDGF and VEGF on the SC cytokine profile in inflammatory and non-inflammatory conditions (**A–D**) Effects of an inflammatory environment on SC-mediated secretion of pro-inflammatory cytokines TNF-α (**A**), IL-6 (**B**) and anti-inflammatory cytokines TGF-β (**C)**, IL-10 (**D**). (**E–H**) Effects of growth factors alone on TNFα (**E**), IL-6 (**F**), TGFβ (**G**) and IL-10 (**H**) cytokine levels. (**I–L**) Effects of growth factors in inflammatory environment on TNF-α (**I**), IL-6 (**J**), TGF-β (**K**) and IL-10 (**L**) cytokine levels. Dashed lines represent the lowest detectable limit of the kit for each cytokine (TNF-α, TGF-β at 8 pg/mL, IL-6 at 4 pg/mL and IL-10 at 32 pg/mL). * p ≤ 0.05, measured using Mann–Whitney U test for (**A–D**) and one-way ANOVA followed by post hoc Dunn’s test for (**E**–**L**). Error bar represents mean ± SEM for three technical replicates of three biological replicates. (**Note:** The y-axis scale of TGF-β on **C**, **G** and** K** are different).

In control medium, SCs did not produce any detectable levels of the pro-inflammatory cytokine TNF-α (Fig. 5A), however, they did produce detectable levels of IL-6 (Fig. 5B) and TGF-β (Fig. 5C). When cultured in the inflammatory environment, SCs produced significantly higher levels of TNF-α and IL-6 (Fig. 5A-B) and continued to produce detectable levels of TGF-β (Fig. 5C), but not IL-10 (Fig. 5D).

Next, we assessed the influence of VEGF and PDGF as a single or combined administration on the production of the cytokines by SCs. We found that both single and combined administration of VEGF and PDGF did not produce any detectable levels of TNF-α (Fig. 5E). This was contrary to IL-6 (Fig. 5F) and TGF-β (Fig. 5G) levels, which were not significantly different compared to control medium. Similar to TNF-α, IL-10, did not produce any detectable levels (Fig. 5H). Thus, we assumed that VEGF and PDGF, applied alone or in combination, had little, if any, effect on cytokine production by SCs.

Ultimately, we wanted to assess whether the growth factor combinations in an inflammatory environment could produce any changes in the expression of the cytokines. When we compared the cytokine levels to that of inflammatory only condition (IFN-γ + LPS), we found that PDGF treatment reduced the production of TNF-α by SCs in the inflammatory environment (Fig. 5I). On the contrary, IL-6 (Fig. 5J), TGF-β (Fig. 5K) and IL-10 (Fig. 5L) levels did not significantly alter under the influence of the growth factors. Thus, both gene expressions and cytokine assay complemented each other, and we concluded that PDGF can reduce the risk of further damage to surrounding environment by suppressing pro-inflammatory cytokine TNF-α while leaving other cytokines unaltered in an inflammatory environment.

### Effect of VEGF and PDGF on nerve repair associated and myelination associated genes

**Figure 6:**
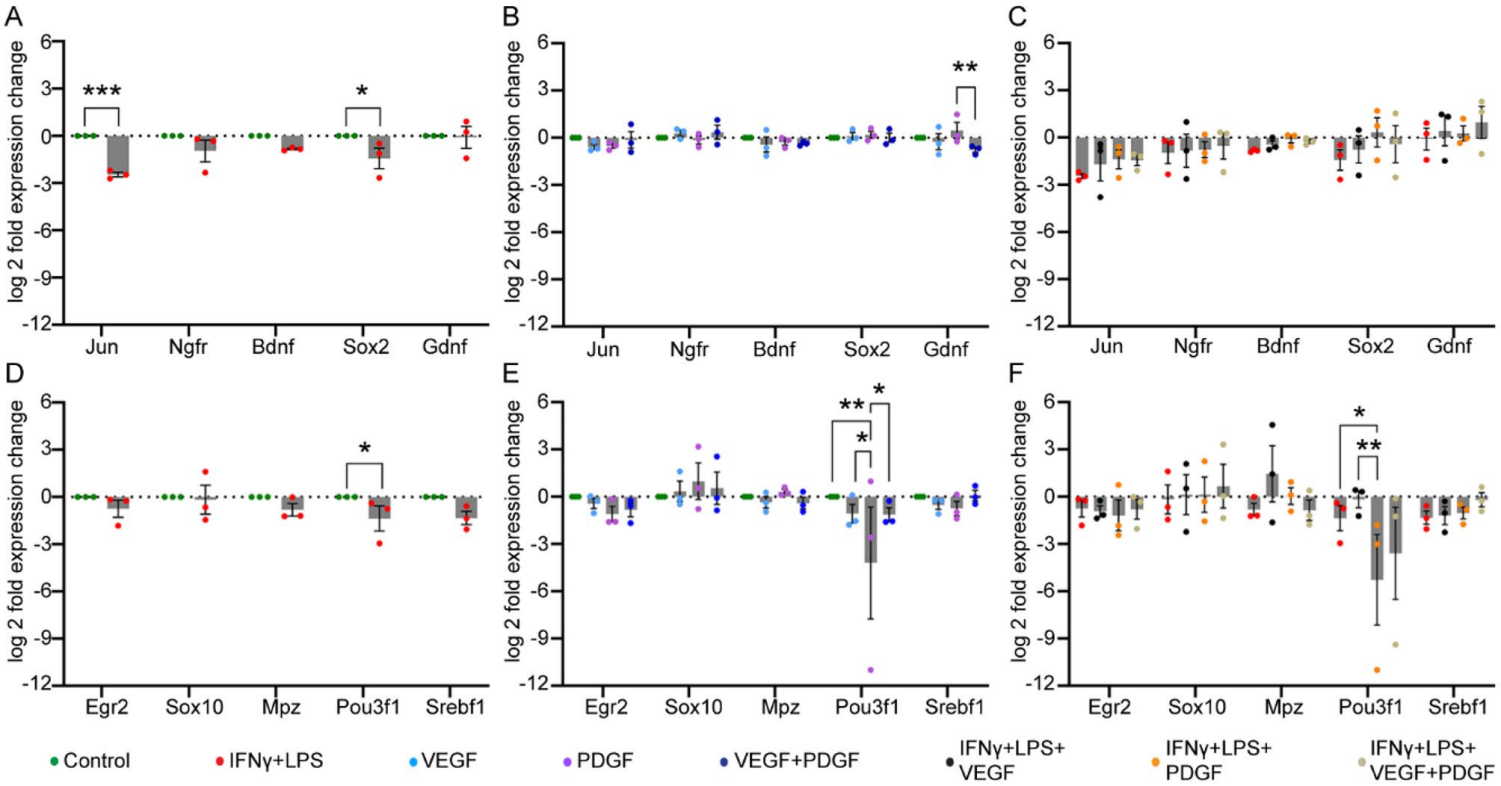
Effects of VEGF and PDGF on the expression of genes associated with nerve repair and myelination in SCs, with and without inflammatory condition. (**A–C**) Bar graphs of log twofold expression changes of nerve repair associated genes in (**A**) untreated control vs inflammatory medium (IFN-γ + LPS), (**B**) control vs VEGF vs PDGF vs combined VEGF + PDGF, (**C**) VEGF, PDGF, combined VEGF + PDGF in inflammatory medium. (**D–F**) myelination associated genes of (**D**) control vs inflammatory medium (IFN-γ + LPS), (**E**) control vs VEGF vs PDGF vs combined VEGF + PDGF, (**F**) VEGF, PDGF, combined VEGF + PDGF in inflammatory medium. Asterisk represents * p ≤ 0.05, ** p < 0.01, *** p < 0.001, all were measured using two-way ANOVA followed by post hoc Fisher’s LSD test. Error bar represents mean ± SEM for three biological replicates.

The two most important functions of SCs are to aid in nerve repair^46^ and expressing myelin in the presence of stimulators^47^. Therefore, in our next aim we wanted to see the influence of VEGF and PDGF, in the absence or presence of inflammatory environment, on expression of genes associated with nerve repair (Jun, Ngfr, Bdnf, Sox2 and GDNF) and myelin expression (Egr2, Sox10, Mpz, Pou3f1 and Srebf1). First, we assessed the effects of an inflammatory environment on the expression of the genes represented in the former group. We found that in an inflammatory environment (IFN-γ** + **LPS), expression of the nerve repair associated genes, Jun and Sox2, was significantly downregulated compared to control (baseline) (Fig. 6A). Next, we wanted to observe the influence of the growth factors on these genes without any inflammatory mediators. The growth factors, alone or combined, had negligible effect on the expression of these genes compared to control. Nevertheless, we noticed a significant difference in GDNF expression between PDGF and combined VEGF and PDGF treatment (Fig. 6B). When we added these growth factors in inflammatory medium, no obvious changes in the expression pattern were observed, compared to control. Additionally, the growth factors did not produce any significant expression changes compared to inflammatory medium alone (Fig. 6C).

We next assessed the expression of myelination associated genes. While inflammatory medium showed a downregulating trend of all the genes compared to control, only Pou3f1 (Oct6) showed significant changes (Fig. 6D). When we introduced the growth factors, all of them had negligible alterations in expression compared to control, except for Pou3f1 where PDGF significantly downregulated the gene compared to control, VEGF and combined VEGF and PDGF (Fig. 6E). Next, when we introduced the growth factors in inflammatory medium, PDGF showed significant difference in Oct6 expression between VEGF and inflammatory only medium (Fig. 6F).

Thus, we concluded that inflammation triggered the downregulation of genes from both the groups (Jun, Sox2 and Pou3f1). Amongst the growth factors, PDGF was only able to downregulate the myelin associated gene, Pou3f1. The latter property was also observable in inflammatory state where it showed significant downregulation compared to VEGF and inflammatory only medium. In contrary, no significant alterations were observable with combined VEGF and PDGF.

## Discussion

In the present study, we demonstrated the immunomodulatory and neuroprotective properties of SCs under the influence of VEGF and PDGF in vitro*.* Our first aim was to see how the growth factors affected the proliferation of SCs. Both VEGF and PDGF are known angiogenic factors and have well documented neurotrophic activities^17,36,48^. We successfully replicated previous findings based on the dose-dependent proliferation of SCs when exposed to VEGF and PDGF, individually^36,37^. To our knowledge, the effect of combined VEGF and PDGF on SCs has not been reported previously. Interestingly, addition of combined VEGF and PDGF, at doses 40 ng/mL and above, led to increased proliferation (shown by increased EdU uptake in p75NTR positive cells) and metabolic activity. We also assessed the role of the growth factors on the proliferation of non-SC cells present in the mixed culture. We did not notice any remarkable outcome on their proliferation except for combined VEGF and PDGF at the higher dose; in fact VEGF (30–50 ng/mL) reduced proliferation. Thus, further investigations are needed to understand the changes on these cells. In contrast, for the SCs which express the corresponding receptors of VEGF and PDGF, the growth factors drove the proliferation of immature SCs^49,50^. We also wanted to see whether inflammatory mediators (IFN-γ + LPS) had any effect on the proliferation and metabolic activity of SCs. The inflammatory mediators alone as well as VEGF and combined VEGF and PDGF, adversely affected the proliferation but not the metabolic activity of the heterogenous population of SCs. Interestingly, PDGF did not lead to a detrimental effect on the SC population in this condition. This is important as it shows that PDGF-treated SCs in inflammatory conditions can benefit in an acute injury site. However, mere survival of the cells is not sufficient for a successful transplantation strategy. As the overall goal is to understand how SCs can be used for transplantation, we used primary cultures of SCs (purity > 60% based on p75NTR expression, > 80% based on DsRed expression), which also contained other cells. Thus, the responses observed are to be considered within this context.

Myelin is a known inhibitor of regeneration and improper clearance is related to delayed SCI healing^34^. Thus, in our next aim we wanted to see how effectively SCs could clear myelin debris in an inflammatory environment. We observed that the pro-inflammatory environment greatly reduced the efficiency of clearance of myelin debris by SCs in the early and intermediate stages. Hence, we wanted to see if VEGF, PDGF or their combination could improve the clearance of myelin debris since both VEGF^51^ and PDGF^52^ have been linked with enhanced autophagy in SCI. We first demonstrated that PDGF showed an increased phagocytic trend in a shorter period than either VEGF or combined VEGF + PDGF, in the absence of any inflammation. This could be due to higher proliferation of SCs by PDGF, however, other mechanisms are likely to be involved since VEGF was unable to phagocytose myelin debris despite also increasing the proliferation of SCs. When we further challenged the SCs with the growth factors in the pro-inflammatory environment, PDGF still maintained a greater efficacy for stimulating cells to be phagocytic of myelin debris. Surprisingly, both VEGF and combined VEGF + PDGF in inflammatory mediators also enhanced phagocytosis.

We believe that the above pattern (single vs combination growth factor application in metabolic and phagocytic activity) exists due to the differential activation of downstream signalling in SCs. In SCs, phagocytic clearance of myelin debris is influenced by TAM receptors (Tyro 3, Axl, Mertk)^34^. Following SCI, the Erk pathway is activated which is further responsible for Gas6-TAM receptor activation^53,54^; the Erk pathway is also activated by PDGF for differentiation^39^. However, SCI can also lead to upregulation of VEGFR-1 in SCs^55^, with VEGFR-1 having high affinity for VEGF_165_ (VEGF-A)^56^, which acts through the PI3k, an Erk-opposing downstream signalling pathway^39,57^. Thus, we can hypothesize that PDGF by itself would enhance phagocytosis including the metabolic activity by activating TAM receptors, but when used in combination with VEGF, there can be a subdued outcome due to the balance in opposing (Erk vs PI3K) downstream signalling of pathways. Nevertheless, while these growth factors can stimulate myelin phagocytosis in vitro, the efficacy needs to be confirmed in vivo.

Following SCI, pro-inflammatory cytokines like TNF-α and IL-6 lead to a more deleterious acute phase of SCI^58^. A previous study had shown that transplantation of SCs reduced the recruitment of pro-inflammatory macrophages and microglia with consequent upregulation of the anti-inflammatory phenotype, however the cytokine profile remained unknown^59^. Therefore, our next aim was to determine if the growth factors could help in modulation of the secretory cytokine genes. Initially, we established that both the pro-inflammatory cytokine genes were upregulated when challenged by inflammatory conditions without altering the expression of anti-inflammatory genes. Nevertheless, the growth factors by themselves did not alter expression of the genes. However, in inflammatory challenged SCs, our observations revealed that PDGF significantly downregulated the expression of TNF-α gene. To corroborate the corresponding translational changes, we performed a cytokine assay. We first wanted to establish how an inflammatory setting determined the cytokine pattern of SCs. This showed that it significantly raised the levels of pro-inflammatory cytokines TNF-α and IL-6 without any effect on anti-inflammatory cytokines, TGF-β and IL-10. However, VEGF, PDGF and their combination did not elevate the levels of TNF-α and IL-10 above detectable levels but raised the levels of IL-6 and TGF- β, although these were non-significant. Further, when we challenged the SCs with the growth factors in an inflammatory environment, PDGF significantly reduced the level of TNF-α, thereby, demonstrating an additional beneficial role in addition to effective myelin clearance. Thus, based on the above expression pattern we concluded that PDGF-treated SCs, when challenged in an inflammatory environment, provided a beneficial effect in comparison to either VEGF or combined VEGF and PDGF.

In addition to maintenance of wound healing conditions by SCs in the presence of VEGF and PDGF, they are also involved in plasticity by down-regulation of genes promoting myelination (Egr2, Sox10, Mpz, Pou3f1 and Srebf1) with relative up-regulation of immature SCs (de-differentiated) nerve repair associated genes (Jun, Ngfr, Bdnf, Sox2 and GDNF)^53,60^. Thus, our final aim was to see if the growth factors were able to modulate the genetic expressions of the groups involved in nerve repair and myelination. We first challenged the SCs in an inflammatory environment and our findings revealed that the inflammatory condition led to a trend showing downregulation of most of the genes, particularly Jun, Sox2 (nerve repair associated) and Pou3f1 (myelin expression associated). However, Gdnf and Sox10 did not show any alterations with untreated control. Next, we wanted to establish if VEGF and PDGF in the absence of any inflammatory mediator modulated the expression pattern of the genes. We observed that only GDNF was significantly upregulated by PDGF in comparison to combined VEGF + PDGF which could be due to activation of positive feedback following stimulation of Erk pathway by PDGF^61^. Consequently, PDGF also down-regulated Pou3f1 which further led to the conclusion that it could be beneficial in acute injury where nerve repair is a priority, and myelination is more important for maintenance of the axon and its functionality^62^. Further, PDGF when challenged in an inflammatory environment also downregulated Pou3f1 compared to inflammatory only and VEGF. Thus, PDGF showed beneficial role in both baseline and challenging environment for SCs.

In conclusion, as SCI initiates a pro-inflammatory microenvironment in the first few days, transplantation of cells that can ameliorate the injury site needs to be considered within the context of the cell responses within an inflammatory environment. Combined VEGF and PDGF has previously been used in animal studies to partially repair SCI but improvements are still left to be explored. Our current study demonstrated that an inflammatory environment affects SCs, but that the addition of the growth factors can also stimulate the phagocytosis of myelin debris by SCs and can modulate the expression of cytokines and gene expression. However, it is noteworthy that most of the assays do contain a substantial number of cells which do not express S100β-DsRed or are not p75NTR immunopositive. Thus, this pattern reflects an overall response of the heterogenous population which is predominantly SCs. In this context, a crosstalk between these cells and SCs can become very vital^63^. Hence, further strategies (purification or enrichment) are required to establish the effects of VEGF and PDGF on this population; however whether a pure population of SCs responds in a way that reflects in vivo responses is yet to be determined. Overall, our observations suggested that PDGF had a prominent phenotypic and genotypic effect. Additionally, combined VEGF and PDGF also produced beneficial outcomes (proliferative, metabolic and phagocytic effect) but further co-culture studies are required to confirm their role. Thus, this study may aid the development of a potential combined therapy using SC transplantation with exogenous VEGF and PDGF application.

## Methods

### Ethics

All procedures were approved by Griffith University’s Animal Ethics Committee (MSC/13/18/AEC) and by the University Biosafety Committee (NLRD/003/2020_Var2) under the guidelines of the Australian Commonwealth Office of the Gene Technology Regulator Regulator and the National Health and Medical Research Council of Australia. All authors complied with the ARRIVE guidelines.

### Reagents

To create a representative pro-inflammatory condition in vitro, we used the combination of interferon-γ (IFN-γ) (1 ng/mL, Abcam) and lipopolysaccharide (LPS) from *E. coli* O111:B4 (100 ng/mL, Sigma) as suggested previously^64^. To evaluate the activity of growth factors, we used human VEGF recombinant protein (Gibco) and mouse PDGF-BB recombinant Protein (Gibco).

### Primary cell culture

Primary Schwann cell culture was prepared from S100β-DsRed transgenic mice, as previously described^38,65,66^. Briefly, each biological replicate used 9–12 pups of age postnatal day 7–9; pups were decapitated, and a sagittal section was made after removing the jaw. The trigeminal ganglia were isolated, and the explants were transferred to a 24-well culture plate. The explants were allowed to attach in complete culture medium, comprising Dulbecco’s Modified Eagle Medium (DMEM), 10% fetal bovine serum, gentamycin (50 μg/mL) and GlutaMAX™ (200 μM, Gibco). Following this, a half media change was done every alternate day, until confluency was reached. The cells obtained from this process comprised 80–85% DsRed-positive cells. Further, they were immunostained with a glial cell marker, rabbit anti-p75NTR antibody (1:1000, CST) which showed 61.2% of positive cells. The cultured cells were then used for further assays. Thus, one biological replicate indicates cells obtained from 9 to 12 pups.

### Viability and EdU proliferation assay

SCs were seeded at a density of 4000 cells/well in 384-well microplate (Greiner) for overnight incubation in complete culture media. The following day, the media was replaced by serum-free media with increasing concentrations of human VEGF recombinant protein, mouse PDGF-BB recombinant protein from 0 to 100 ng/mL, as single and combined dose. After 20 h of incubation, 5 µL of resazurin (500 µM, Sigma) was added to each well to reach to a final concentration of 50 µM. Following this, the plate was incubated for further 4 h at 37 °C and 5% carbon dioxide (CO_2_). The fluorescent signal of the metabolized component, resorufin, was then quantified using POLARstar Omega (BMG Labtech) plate reader at (584/590-10). For EdU proliferation assay, Click-iT EdU Cell Proliferation Kit for Imaging, Alexa Fluor 647 dye (Thermofisher) was used, and manufacturer’s protocol was followed with slight modification. Similar conditions were maintained, as mentioned previously, with addition of EdU labelling solution (2 mM) for 24 h. The plate was then fixed and stained with p75NTR antibody to identify EdU and p75NTR positive cells. Images were acquired using Nikon Ti2 widefield microscope and approximately 160 cells (~ 60% p75 positive cells) were counted from each field of view (FOV). A total of 36 FOV were used per technical replicate and further processing was done with Cell Profiler 4.2.1 software. Representative images were taken using Olympus FV3000 confocal microscopy at 20X objective.

### Myelin debris preparation and confirmation by Western blot

Myelin was prepared following a previously established protocol^67^. Briefly, 10–12 S100β-DsRed transgenic mice (12 months old) were euthanased and brains were dissected out and homogenized in 0.32 M sucrose solution (diluted from 1 M sucrose solution in Tris–Cl buffer). The homogenate was carefully layered on top of 0.83 M sucrose solution and ultracentrifuged at 100,000×g for 45 min at 4 °C. Further, myelin debris was pipetted out from the interface of the two layers, re-homogenized and washed in Tris–Cl buffer for three rounds. Finally, the pellet was washed in PBS stored at a concentration of 50 mg/mL at −80 °C. Purity of the debris was assessed by Western Blot. Protein was extracted using pre-extraction buffer (Nuclear Extraction Kit, Abcam) with 0.2% TritonX-100 and measured using Pierce BCA protein assay kit (Thermo Scientific). It was then run in Bolt 10%, Bis–Tris, 1.0 mm, Mini Protein Gel and transferred to a polyvinylidene membrane using iBlot 2 Gel transfer device (Thermofisher). Further, blocking was done with 5% skim milk-0.2% Tween 20-PBS solution for 1 h and incubated with anti-beta actin (1:1000, Abcam) antibody and anti-myelin basic protein (MBP) antibody (1:1000, GeneTex) overnight at 4 °C. The following day, the membrane was washed three times and incubated with anti-mouse and -rabbit HRP antibody (1:2000, Thermofisher) for 1 h at room temperature. The membrane was then washed, incubated with Immobilon Western Chemiluminescent HRP substrate, and imaged under ChemiDoc MP gel imaging system (BioRad). To check for the presence of contaminating synaptosomes, the membrane was further incubated with anti-PSD95 antibody (1:1000, GeneTex). Extracted synaptosome was used as a positive control for anti-PSD95 antibody (Supplementary Fig. S3).

### Phagocytosis assay of myelin debris

For phagocytosis assays, SCs were seeded at 4000 cells/well and incubated overnight. The following day, myelin debris was labelled with pHrodo Green STP Ester dye (pHrodo STP; Thermofisher), as per manufacturer’s instructions. The pHrodo STP stock (2 mg/mL; 2 mM) was diluted to 12.5 µM concentration in labelling buffer (0.1 M NaHCO_3_ buffer; pH 8.3). The diluted myelin stock was washed 4–5 times and resuspended in PBS. The pHrodo STP-labelled myelin debris was used at 1 mg/mL per condition. For phagocytosis evaluation, live cell imaging was performed using IncuCyte live cell imaging system (Sartorius). As the SCs express DsRed protein and myelin debris was labelled with pHrodo green STP dye, they were exposed at 150 ms in the red and green channel, respectively, for individual fluorophore emission visualization. Images were acquired every 30 min and analysis of phagocytosing cell was performed using Cell profiler 3.1.9 software.

### Immunofluorescence and confocal microscopy

After phagocytosis assay, the supernatant was removed, cells with debris were then fixed with 4% PFA for 10 min and washed with PBS three times for 5 min each. Images were acquired using Olympus FV3000 confocal microscopy at 20X objective. Z-stacking was done to locate debris inside the cell. This was followed by 3D rendering using Imaris 9.4 software (Supplementary Video S4).

### Enzyme-linked immunosorbent assay (ELISA) of cytokines

For extraction of cytokines from supernatant, SCs were seeded at 6 × 10^5^ cells/well in a 6-well plate. They were allowed to grow until they reached 80% confluency. Following this, the complete media was replaced with serum-free activated media containing growth factors (VEGF, PDGF, VEGF + PDGF) and no treatment (Control). To determine the cytokines in inflammatory conditions, the following groups were used: (i) IFN-γ + LPS, (ii) IFN-γ + LPS + VEGF, (iii) IFN-γ + LPS + PDGF, and (iv) IFN-γ + LPS + VEGF + PDGF. We collected the supernatants after 24 h treatment and then centrifuged the same at 1500 rpm for 10 min at 4 °C. The supernatants were stored at −80 °C, until further analysis. We ensured that the supernatants were used as neat, or 1 in 10, or 1 in 100 dilutions and took readings which were within the detectable range of the kit. We used four different cytokine kits for our sandwich ELISA assays: (1) pro-inflammatory: IL-6 (Invitrogen) and TNF-α (Invitrogen) and (2) anti-inflammatory: IL-10 (Invitrogen) and TGF-β (Invitrogen). The entire protocol was followed as per manufacturer’s instructions. Plate readings were taken using absorbance values at 450 nm and 570 nm under POLARstar Omega plate reader.

### Real time quantitative polymerase chain reaction (qPCR)

For qPCR assays, 4 × 10^5^ cells/well were seeded in a 24-well microplate in complete media for overnight incubation in 5% CO_2_ and at 37 °C. The next day, the media was replaced with serum-free activated media with conditions, as mentioned above. After 24 h of incubation, the media was completely removed, washed with 1 × PBS and the cells were lysed using RNA lysis buffer (PureLink RNA minikit, Invitrogen) with 1% mercaptoethanol. The samples were stored at −80 °C until further processing. Further processing was done after homogenizing, washing with 70% ethanol and centrifuged at 12,000×g for 15 s. The final elution step was completed by centrifuge using 30 µL of UltraPure DNase/RNase-Free distilled water. Quantity and quality of RNA was measured using NanoDrop 1000. cDNA was produced using SuperScript IV VILO Master Mix with ezDNase Enzyme (Invitrogen). PCR reaction was run using PowerUp SYBR green Master mix (2X, Thermofisher) in a Quantstudio 6 flex lightcycler machine (Thermofisher). For each qPCR reactions, we used 500 nM of Forward and Reverse primers each (Supplementary Table S5) with 20 ng of cDNA. T_m_ of all the primers were above 60 °C. Settings for the PCR reaction were as follows: (a) Uracil DNA-glycosylase activation at 50 °C for 2 min, (b) Dual-Lock DNA polymerase at 95 °C for 2 min, (c) denaturation at 95 °C for 15 s with annealing and extension at 60 °C for 1 min repeated for 40 cycles. Melt curve was also generated to note for any primer-dimer formation.

### Statistical tests and graphs

All data were expressed as the mean ± standard error of the mean (SEM). Comparisons of two groups were done by unpaired *t*-test (two-tailed) with Welche’s correction or Mann–Whitney *U* test, as where appropriate. For qPCR assays, log2-fold change of gene expression was evaluated. For multiple groups, either one-way or two-way analysis of variance (ANOVA) was used, as appropriate, and was followed by a post-hoc test comprising of Dunnett’s, Dunn’s or Fisher’s LSD test, respectively. We used GraphPad prism 9 for statistical evaluations and graphical representations. Statistical significances were represented in each figure, separately.

## Supplementary Information

Supplementary Information 1.

Supplementary Video 1.
