# Author Correction: In vitro modulation of Schwann cell behavior by VEGF and PDGF in an inflammatory environment

**DOI:** 10.1038/s41598-022-12927-6

**Published:** 2022-05-24

**Authors:** Souptik Basu, Indra N. Choudhury, Lynn Nazareth, Anu Chacko, Todd Shelper, Marie-Laure Vial, Jenny A. K. Ekberg, James A. St John

**Affiliations:** 1 Clem Jones Centre for Neurobiology and Stem Cell Research, Griffith University, Nathan, QLD Australia; 2 Menzies Health Institute Queensland, Griffith University, Southport, QLD Australia; 3 Griffith Institute for Drug Discovery, Griffith University, Nathan, QLD Australia

Correction to:* Scientific reports* 10.1038/s41598-021-04222-7, published online 13 January 2022

The original version of this Article contained an error in Figure 5, where the graph for panel (L) was incorrect. The original Figure 5 and accompanying legend appear below.

**Figure 5:**
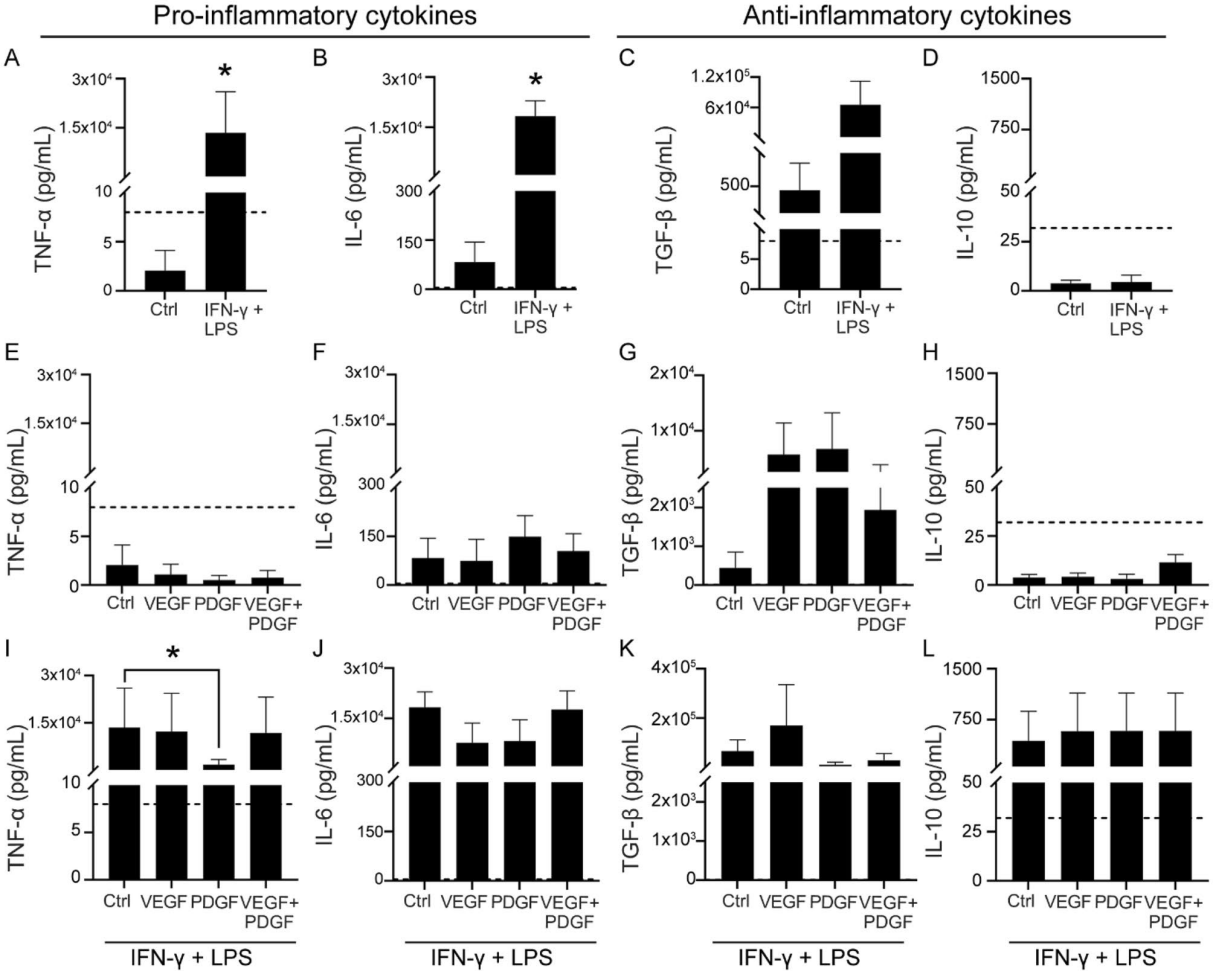
Effects of PDGF and VEGF on the SC cytokine profile in inflammatory and non-inflammatory conditions (**A–D**) Effects of an inflammatory environment on SC-mediated secretion of pro-inflammatory cytokines TNF-α (**A**), IL-6 (**B**) and anti-inflammatory cytokines TGF-β (**C)**, IL-10 (**D**). (**E–H**) Effects of growth factors alone on TNFα (**E**), IL-6 (**F**), TGFβ (**G**) and IL-10 (**H**) cytokine levels. (**I–L**) Effects of growth factors in inflammatory environment on TNF-α (**I**), IL-6 (**J**), TGF-β (**K**) and IL-10 (**L**) cytokine levels. Dashed lines represent the lowest detectable limit of the kit for each cytokine (TNF-α, TGF-β at 8 pg/mL, IL-6 at 4 pg/mL and IL-10 at 32 pg/mL). *p ≤ 0.05, measured using Mann–Whitney U test for (**A–D**) and one-way ANOVA followed by post hoc Dunn’s test for (**E**–**L**). Error bar represents mean ± SEM for three technical replicates of three biological replicates. (Note: The y-axis scale of TGF-β on **C**, **G** and **K** are different).

The original Article has been corrected.

