# Supplementary material for: In vitro modulation of Schwann cell behavior by VEGF and PDGF in an inflammatory environment
Source: Sci Rep. 2022 Jan 13;12:662. doi: 10.1038/s41598-021-04222-7 (PMC8758747; doi:10.1038/s41598-021-04222-7)
Supplement: Supplementary file 1 — Supplementary Information 1. [file 41598_2021_4222_MOESM1_ESM.pdf]

Supplementary information

Supplementary Figure S1

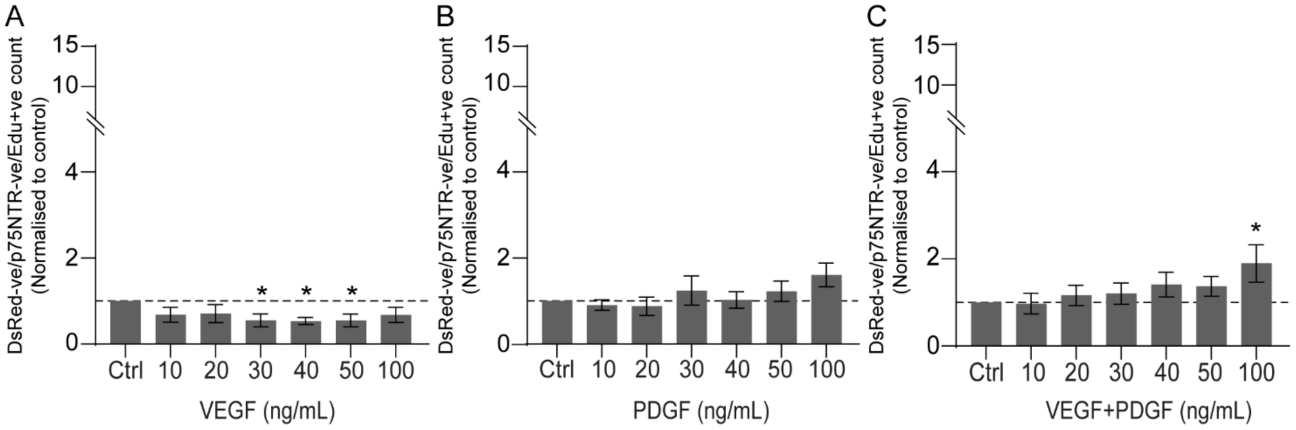

Supplementary Figure S1. Proliferation of DsRed -ve/ p75NTR -ve/ EdU +ve cells. (A) VEGF, (B) PDGF, (C) VEGF+PDGF. Dashed lines show normalisation of EdU count to control (set at 1). * represents p<0.05 measured using one way ANOVA with post hoc Dunn's test. Error bar represents mean ± SEM for three biological replicates.

Supplementary Figure S2

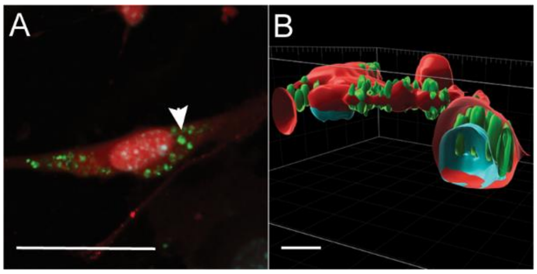

Supplementary Figure S2. Myelin debris within SCs. **(A)** Confocal microscope image showing myelin debris (green; white arrow) inside a DsRed-expressing SC (red). Scale bar: 40 µm. (**B**) 3D rendering (obtained using using Imaris 9.4) showing myelin (green) inside a DsRed-expressing SC. Hoechst nuclear stain (cyan). Scale bar: 15 µm.

Supplementary Figure S3

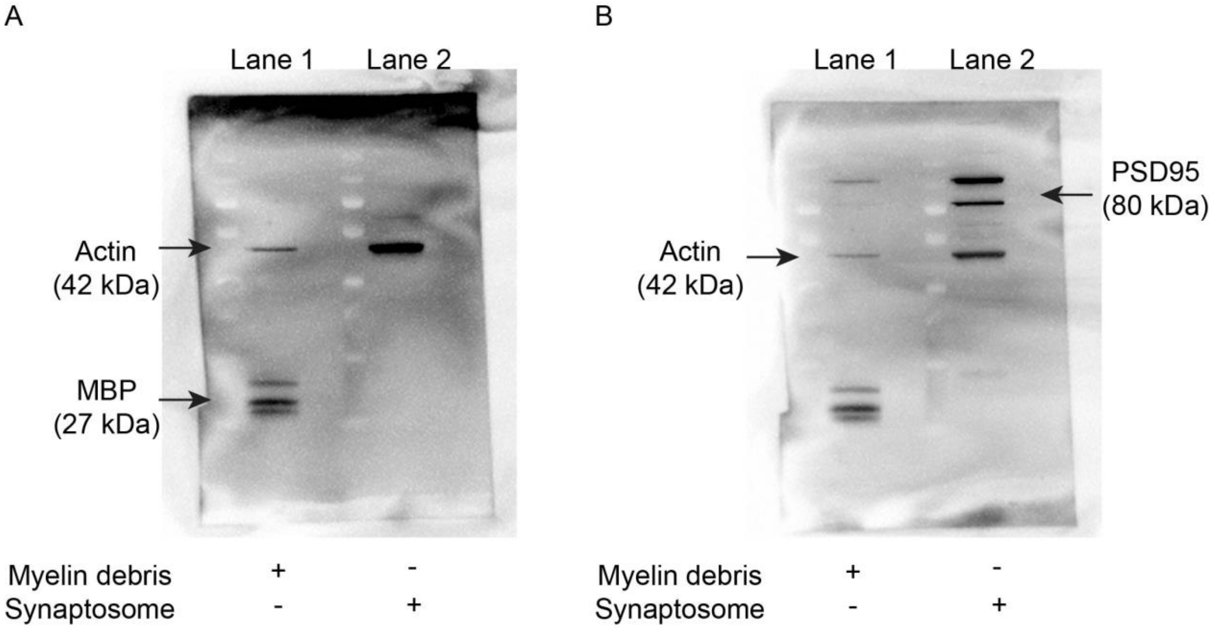

Supplementary Figure S3. Western blot of extracted myelin debris and synaptosome. (**A**) Lane 1: for myelin debris protein and Lane 2: for synaptosomal protein. MBP stains at 27 kDa with loading control Actin at 42 kDa. Actin stains in both Lane 1 and 2. But MBP only stains with myelin debris showing negligible contamination. (**B**) Lane 1: for myelin debris protein and Lane 2: for synaptosomal protein. PSD95 stains at 80 kDa with loading control Actin at 42 kDa. However, no stain was observed at 27 kDa, showing the specificity of the antibody.

Supplementary Video S4. 3D rendering of phagocytosed myelin debris (green spots) in DsRed SCs (red object) using Imaris 9.4 software. Scale bar varies according to magnification as mentioned in the left bottom corner of the video.

Supplementary Table S5.

Gene Name	Forward sequence	Reverse sequence	Ref.
Tnfa	GCCTCTTCTCATTCCTGCTTG	CTGATGAGAGGGAGGCCATT	1
Tgfb1	AAGTTGGCATGGTAGCCCTT	GCCCTGGATACCAACTATTGC	2
Il6	CCAGTTGCCTTCTTGGGACT	GGTCTGTTGGGAGTGGTATCC	2
Il10	GCTCTTACTGACTGGCATGAG	CGCAGCTCTAGGAGCATGTG	2
Jun	GAACTGCATAGCCAGAACAC	GTTGAAGTTGCTGAGGTTGG	3
Sox2	GCCATTAACGGCACACTGC	CCCCTCCCAATTCCCTTGTA	3
Ngfr	AGCCCTCAAGGGTGATGGC	CCTCGTGGGTAAAGGAGTCTATATG	4
Bdnf	TGCAGGGGCATAGACAAAAGG	CTTATGAATCGCCAGCCAATTCTC	5
Gdnf	TGACCAGTGACTCCAATATGCC	CCGCTTGTTTATCTGGTGACCT	6
Egr2	AGGCCCCTTTGACCAGATGA	AAGATGCCCGCACTCACAAT	3
Pou3f1	CGCCAAGCAGTTCAAGCAA	TTGAGCAGCGGTTTGAGCTT	3
Sox10	TCAAGAAGGAACAGCAGGAC	CTTTCGTTCAGCAACCTCCAG	3
Mpz	CCCTGGCCATTGTGGTTTAC	CCATTCACTGGACCAGAAGGAG	3
Srebf1	CCTGCTTGGCTCTTCTCTTT	CTGGTGCAGCTTATGGTAGAC	7

Supplementary Table S5. Forward and reverse primer sequences used in the qPCR reactions.

References for primers

1. Yamakawa, I. et al. Inactivation of TNF-α ameliorates diabetic neuropathy in mice. American Journal of Physiology-Endocrinology and Metabolism (2011).
2. Shaul, M. E., Bennett, G., Strissel, K. J., Greenberg, A. S. & Obin, M. S. Dynamic, M2-like remodeling phenotypes of CD11c+ adipose tissue macrophages during high-fat diet–induced obesity in mice. Diabetes 59, 1171-1181 (2010).
3. Hackett, A. R., Strickland, A. & Milbrandt, J. Disrupting insulin signaling in Schwann cells impairs myelination and induces a sensory neuropathy. Glia 68, 963-978 (2020).
4. Wang, Y. et al. p75NTR−/− mice exhibit an alveolar bone loss phenotype and inhibited PI3K/Akt/β-catenin pathway. Cell proliferation 53, e12800 (2020).
5. Huang, T. & Krimm, R. F. Developmental expression of Bdnf, Ntf4/5, and TrkB in the mouse peripheral taste system. Developmental Dynamics 239, 2637-2646 (2010).
6. Allardyce, H. et al. Renal pathology in a mouse model of severe Spinal Muscular Atrophy is associated with downregulation of Glial Cell-Line Derived Neurotrophic Factor (GDNF). Human molecular genetics 29, 2365-2378 (2020).
7. Monnerie, H. et al. Reduced sterol regulatory element-binding protein (SREBP) processing through site-1 protease (S1P) inhibition alters oligodendrocyte differentiation in vitro. Journal of neurochemistry 140, 53-67 (2017).
